# Development and Validation of an HPLC Method for Determination of Antidiabetic Drug Alogliptin Benzoate in Bulk and Tablets

**DOI:** 10.1155/2018/1902510

**Published:** 2018-09-24

**Authors:** Hani Naseef, Ramzi Moqadi, Moammal Qurt

**Affiliations:** ^1^Samih Darwazah Institute for Pharmaceutical Industries, Faculty of Pharmacy, Nursing and Health Professions, Birzeit University, Birzeit, State of Palestine; ^2^Pharmacy Department, Faculty of Pharmacy, Nursing and Health Professions, Birzeit University, Birzeit, State of Palestine

## Abstract

Alogliptin benzoate, a member of dipeptidyl peptidase-4 inhibitors, is a recent drug developed by Takeda Pharmaceutical Company for the treatment of Type 2 diabetes; it potentiates the effect of incretin hormones through the inhibition of their degradation. Alogliptin can be used alone or in combination therapy. A new sensitive and rapid HPLC method was developed for the determination of alogliptin benzoate in bulk and pharmaceutical dosage forms; it was validated according to ICH and FDA guidelines. The HPLC analysis was performed on the Agilent 1200 system equipped with a Hypersil Gold Thermo Scientific C18 (250 cm × 4.6 mm) 5 *µ*m column, with a mixture of acetonitrile and ammonium carbonate buffer in the ratio of 55 : 45 v/v as the mobile phase, at the flow rate of 1.0 mL/min. The detection was performed at the wavelength (*λ*) of 277, and the retention time of alogliptin benzoate was around 4 min. The total run time was 6.0 min. The calibration plot gave linear relationship over the concentration range of 85–306 *µ*g/ml. The LOD and LOQ were 0.03 and 0.09 *μ*g, respectively. The accuracy of the proposed method was determined by recovery studies and was found to be 100.3%. The repeatability testing for both standard and sample solutions showed that the method is precise within the acceptable limits. RSD% of the determination of precision was <2%. The results of robustness and solutions stability studies were within the acceptable limits as well. The proposed method showed excellent linearity, accuracy, precision, specificity, robustness, LOD, LOQ, and system suitability results within the acceptance criteria. In addition, the main features of the developed method are low run time and retention time around 4 min.

## 1. Introduction

Since the first evidence about a known case of diabetes mellitus nearly 3000 years ago and despite the great deal of research that has been done recently, diabetes mellitus is still a wide spread serious disease that affect the life quality of millions of people worldwide. It is estimated that the number of patients with diabetes mellitus will rise to about 592 millions by the year 2035 [1, 2].

It was until the year 1936 that diabetes mellitus was distinguished to Type 1 and Type 2 [1]. Two main features of Type 2 diabetes mellitus is the increased cell resistance to insulin and the dysfunction of the insulin-producing cell in the pancreas (*β*-cells) [2, 3]. The first line of therapy for the treatment of Type 2 diabetes is metformin, but as the disease progresses, a drug combination may be a must [4].

**Figure 1:**
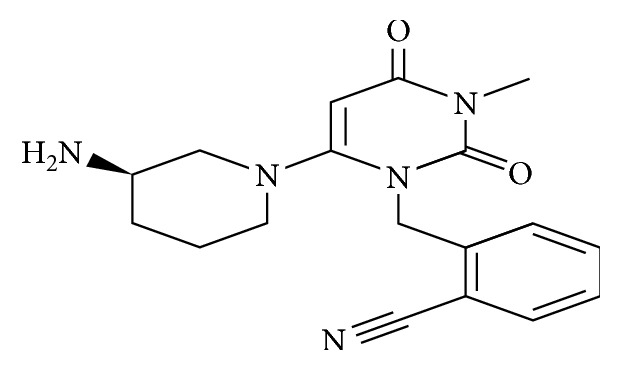
Chemical structure of alogliptin.

Incretin hormones are secreted in response to eating food from the gastrointestinal tract to the blood stream and can stimulate insulin secretion and help control glucose levels; that is, they prepare the body against increase in blood glucose. These hormones include glucagon-like peptide-1 and glucose-dependent insulin tropic polypeptide [5, 6]. Dipeptidyl peptidase-4 is an enzyme found in the human body that helps inactivate the incretin hormones, thus terminating their hypoglycemic effect [2]. Alogliptin a member of dipeptidyl peptidase-4 inhibitors is a recent drug developed in 2010 by Takeda Pharmaceutical Company [2, 7], which is used for the treatment of Type 2 diabetes, and it potentiates the effect of incretin hormones through inhibition of their degradation by the dipeptidyl peptidase-4 enzyme [2, 4]. Alogliptin can be used alone or in combination therapy, and it is now approved in the USA and Europe also [5]. Alogliptin is 2-({6-[(3R)-3-aminopiperidin-1-yl]-3-methyl-2,4-dioxo-1,2,3,4-tetrahydropyrimidin-1yl}methyl)benzonitrile (C18H21N5O2), and its structure is shown in Figure 1 [8].

Analytical method validation ensures that various HPLC analytical techniques shall give reliable and repeatable results; it is a crucial step in developing new dosage forms as it provides information about accuracy, linearity, precision, detection, and quantitation limits. According to the ICH guideline, “the objective of validation of an analytical procedure is to demonstrate that it is suitable for its intended purpose.” It is now obligatory in the process of drug development to supply the validation data for the responsible authorities. Guidelines for analysis method validation include ICH and USP guidelines [9–12].

Literature survey revealed a few methods reported for determination of alogliptin benzoate in bulk drug as well as pharmaceutical preparation [2, 5, 13–16].

In this research, a new sensitive and rapid HPLC method was developed for the determination of alogliptin benzoate in pharmaceutical dosage forms, and this method was validated according to ICH and FDA guidelines.

## 2. Materials and Methods

### 2.1. Instrumentation

Agilent 1200 HPLC system was used for liquid chromatography method development and validation (Santa Clara, USA), equipped with a pump (model G1312A), an auto sampler (ALS) (model G1329A), and a Hypersil Gold Thermo Scientific C18 (250 cm × 4.6 mm) 5 *µ*m column (Paisley, UK), and the detector consisted of UV/VIS operated at 277 nm. Chemstation Software (Version Rev B.04.03 (16)) was used for data processing and evaluation.

### 2.2. Chemicals and Reagents

A pharmaceutical grade sample of alogliptin benzoate (assigned purity 99.4%) was obtained as gift from Jordan Hikma Pharmaceuticals (Amman, Jordan). NESINA tablets containing 8.5 mg alogliptin benzoate were purchased from the local market. Acetonitrile HPLC grade and ammonium carbonate were purchased from Merck (Merck Serono Amman, Jordan). The double distilled water was obtained from a local pharmaceutical company.

### 2.3. Chromatographic Conditions

The mobile phase was prepared by dissolving 1.0 gm ammonium carbonate in 1000 ml water. From the previous solution, 450 ml was mixed with 550 ml of acetonitrile. Prior to use the mobile phase was filtered through 0.45 *μ*m membrane filters and degassed by sonication for 10 min. The analysis was carried out on an Agilent 1200 series HPLC system. The analytes were conducted on an analytical column C18, 5 *µ*m, 250 × 4.6 mm with a detection wavelength of 277 nm. The operating temperature of the column was set at 30°C. The injection volume was 10 *μ*L, and the flow rate was maintained at 1.0 mL/min. The run time was 6 minutes.

### 2.4. Preparation of Standard Solution

A standard solution of alogliptin benzoate was prepared by dissolving an accurately weighed amount of alogliptin benzoate (42.5 mg, which is equivalent to 31.25 mg alogliptin) in 50 ml of the mobile phase, and then 5 mL of the resulting solution was diluted to 25 mL by the same solvent to obtain a standard solution of alogliptin benzoate (170 *µ*g/ml).

### 2.5. Preparation of Sample Solution

Twenty alogliptin tablets were weighed, triturated in porcelain mortar, and mixed, and the average weight of tablet was calculated. Accurately weighed amount of powder equivalent to 25 mg of alogliptin (34 mg alogliptin benzoate) was transferred completely to a 200 mL volumetric flask, and 150 mL of the mobile phase was added and sonicated for 30 minutes. The volume was completed to mark by the same solvent to obtain a solution of alogliptin benzoate with a concentration of 170 *µ*g/ml. The prepared solution was filtered through 0.45 *μ*m membrane filters.

### 2.6. Method Validation

The method was validated as per ICH and FDA guidelines, and the validation parameters included specificity, linearity, range, accuracy, precision, sensitivity (LOQ and LOD), and robustness [9, 17].

#### 2.6.1. Specificity

Specificity is one of the significant features of HPLC, and it refers to the ability of the analytical method to discriminate between the analyte and the other components in the complex mixture [18]. Specificity of the method was evaluated by injecting 10 *μ*l solutions of standard, sample, blank, and placebo separately.

#### 2.6.2. Linearity

To evaluate the linearity and range of the method, different standard solutions were prepared by diluting the standard stock solution with the mobile phase in deferent concentrations of alogliptin benzoate: 85, 136, 170, 204, 255, and 306 *µ*g/ml, which cover 50%, 80%, 100%, 120%, 150%, and 180% of the target concentration, respectively. Three injections from each concentration were analysed under the same conditions. Linear regression analysis was used to evaluate the linearity of the calibration curve by using the least square linear regression method.

#### 2.6.3. Sensitivity

Limit of detection (LOD)/limit of quantitation (LOQ) of alogliptin benzoate were determined by analysing different solutions of alogliptin benzoate and measuring the signal-to-noise ratio. The limit of detection (LOD) is the concentration that gives a signal-to-noise ratio of approximately 3 : 1, while the limit of quantification (LOQ) is the concentration that gives a signal-to-noise ratio of approximately 10 : 1 with %RSD (*n*=3) of less than 10%.

#### 2.6.4. Accuracy

The accuracy of the assay method was determined by recovery studies at three concentration levels (50%, 100%, and 150%), i.e., 85, 170, and 255 *μ*g/ml, and three samples from each concentration were injected. The percentage recovery of added alogliptin benzoate and RSD were calculated for each of the replicate samples.

#### 2.6.5. Precision

The system precision and method precision (repeatability) of the proposed methods were determined by several measurements of standard solution and sample solution, respectively [19–22]. System precision was established by ten measurements of the standard solution at the 100% concentration levels on the same day. Method precision was established by six assay determinations of the sample solution at the 100% concentration levels on the same day [23]. The RSD of obtained results was calculated to evaluate repeatability results.

#### 2.6.6. Robustness

Robustness of the method was verified by applying minor and deliberate changes in the experimental parameters, for example:

- Column temperature: ±5°C
- Flow rate: ±0.2 mL/min
- Wavelength: ±3 nm
- Mobile phase composition, organic composition ±5%

Change was made to evaluate its effect on the method. Obtained data for each case was evaluated by calculating %RSD and percent of recovery.

#### 2.6.7. Stability of Analytical Solutions

The stability of analytical solutions was determined by analysing the standard and sample preparations at 0 h and after one day in refrigerator and at ambient room temperature 30°C. Three injections from each solution were analysed, and the average of the peak and the RSD were calculated.

## 3. Results and Discussion

### 3.1. Method Development and Optimization

Several physical and chemical properties of alogliptin benzoate were obtained from the literature. The analytical method was developed to select preliminary reversed phase HPLC-UV chromatographic conditions, including detection wavelength, mobile phase, stationary phase, and sample preparation procedure. For this purpose, a series of trials were performed by varying the ratio of acetonitrile and ammonium carbonate buffer and optimizing the chromatographic conditions on the Hypersil Gold Thermo Scientific C18 (250 cm × 4.6 mm) 5 *µ*m column. The results of method optimization are summarized in Table 1.

**Figure 2:**
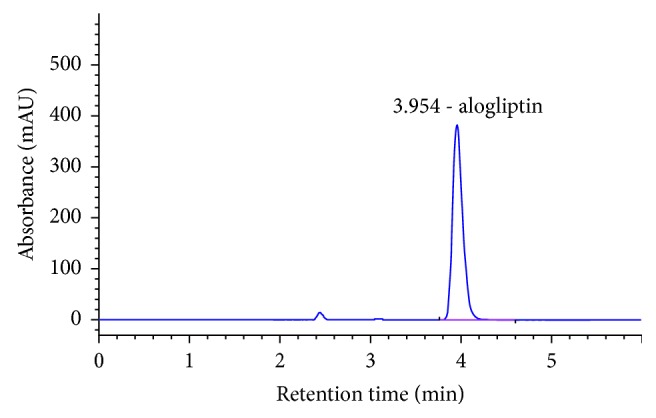
Chromatogram of alogliptin standard solution.

The mobile phase consisting of acetonitrile and ammonium carbonate buffer in the ratio 55 : 45 v/v with a flow rate of 1 mL/min, injection volume 10 *µ*l, run time 6 min, and column temperature 30°C at wavelength (*λ*) 277 was optimized as the best chromatographic conditions for the entire study where alogliptin benzoate was eluted forming symmetrical peak shape, resolution and suitable analysis time with retention time about 4 min (Figure 2).

### 3.2. Method Validation

#### 3.2.1. Specificity

**Figure 3:**
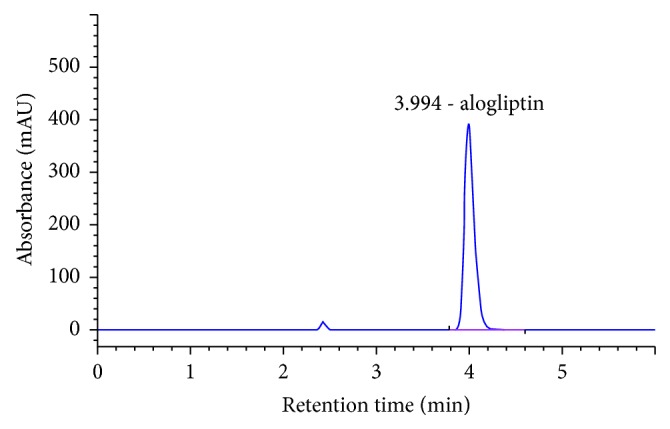
Chromatogram of alogliptin sample solution.

**Figure 4:**
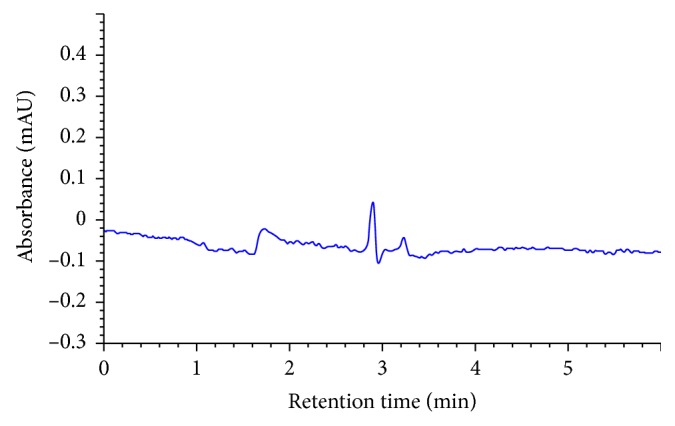
Chromatogram of blank solution.

**Figure 5:**
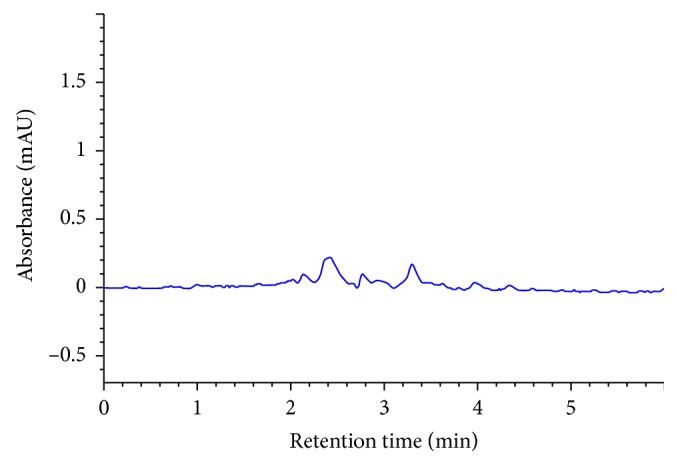
Chromatogram of placebo solution.

Specificity was evaluated by comparing the chromatograms of mobile phase blank, placebo solution, standard solution, and sample solution (alogliptin 170 *μ*g/ml). For this purpose, 10 *μ*l from solutions mobile phase blank, standard solution, and sample solution were injected into the HPLC system separately, and the chromatogram results are shown in Figures 2–5. It can be observed that there no coeluting peaks at the retention time of alogliptin benzoate interference. This result indicates that the peak of the analyte was pure and this confirmed the specificity of the method.

#### 3.2.2. Linearity and Range

**Figure 6:**
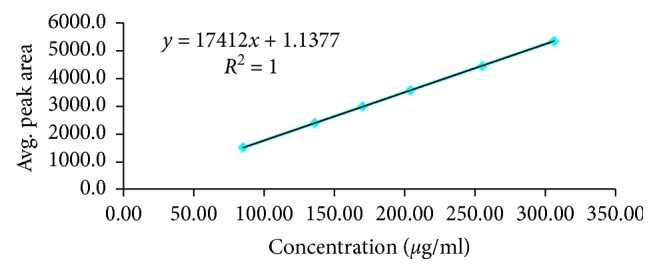
Standard calibration curve of alogliptin benzoate.

Analytical method linearity is defined as the ability of the method to obtain test results that are directly proportional to the analyte concentration, within a specific range. The mean peak area obtained from the HPLC was plotted against corresponding concentrations to obtain the calibration graph. The results of linearity study (Figure 6) gave linear relationship over the concentration range of 85–306 *µ*g/ml for alogliptin benzoate. From the regression analysis, a linear equation was obtained: *y* = 17412*x* + 1.1377, and the goodness-of-fit (*r*^2^) was found to be 1.00, indicating a linear relationship between the concentration of analyte and area under the peak.

#### 3.2.3. Limit of Detection and Limit of Quantification (LOD and LOQ)

The limit of detection (LOD) is the lowest amount of analyte in a sample that can be detected, but not necessarily quantitated, while the limit of quantification (LOQ) is the lowest amount of analyte in a sample that can be quantitatively determined with suitable precision [24]. The results showed an LOD and LOQ for alogliptin of 0.03 and 0.09 *μ*g, respectively.

#### 3.2.4. Accuracy

The accuracy of an analytical procedure expresses the closeness of results obtained by that method to the true value. The results of accuracy showed percentage recovery at all three levels in the range of 99.4–101.9%, and %RDS values were in the range of 0.06–0.43% as shown in Table 2. The results of percentage recovery and %RSD were within the accepted limits from 98.0% to 102.0% and not more than 2.0%, respectively, which indicates the applicability of the method for routine drug analysis.

#### 3.2.5. Precision

The precision of the method is defined as “the closeness of agreement between a series of measurements obtained from multiple sampling of the same homogeneous sample under the prescribed conditions,” and it is normally expressed as the relative standard deviation [25].

The results of both system and method precision showed that the method is precise within the acceptable limits. The RSD, tailing factor, and number of theoretical plats were calculated for both solutions; all the results are within limits. Acceptable precision was not more than 2.0% for the RSD and the tailing factor and not less than 1000 for number of plates, as shown in Tables 3 and 4.

#### 3.2.6. Robustness

The analytical method robustness was tested by evaluating the influence of minor modifications in HPLC conditions on system suitability parameters of the proposed method, as mentioned in Section 2.6.6. The results of robustness testing showed that a minor change of method conditions, such as the composition of the mobile phase, temperature, flow rate, and wavelength, is robust within the acceptable limits. The results are summarized in Table 5. In all modifications, good separation of alogliptin benzoate was achieved, and it was observed that the percent of recovery was within acceptable limits and the %RSD is within limit of not more than 2.0%. The tailing factors and number of theoretical plates were found within acceptable limits as well.

#### 3.2.7. Solution Stability

The percent of recovery was within the range of 98.0% to 102.0% and RSD was not more than 2.0%, indicating a good stability of the sample and standard solutions for 24 hr at both conditions. The percent of recovery was within acceptable limits, and the %RSD is within the limit of not more than 2.0%. The tailing factors and number of theoretical plates were found within acceptable limits as well. The results are shown in Table 6.

## 4. Conclusion

In the present research, a fast, simple, accurate, precise, and linear stability-indicating HPLC method has been developed and validated for alogliptin benzoate, and hence it can be employed for routine quality control analysis. The analytical method conditions and the mobile phase solvents provided good resolution for alogliptin benzoate. In addition, the main features of the developed method are short run time and retention time around 4 min. The method was validated in accordance with ICH guidelines. The method is robust enough to reproduce accurate and precise results under different chromatographic conditions.

## Figures and Tables

**Table 1:** Results of method optimization.

Column used	Mobile phase	Flow rate	Wavelength	Observation	Result
Restek C18, 125 × 4.0 mm i.d., 5 *µ*m	(Buffer : methanol) (45 : 55) v/v	1.0 ml/min	216 nm	Poor resolution 1.4	Method rejected
Thermo Scientific C18, 250 × 4.6 mm i.d., 5 *µ*m	(Buffer : acetonitrile) (25 : 75) v/v	1.0 ml/min	277 nm	Poor resolution 1.6	Method rejected
Thermo Scientific C18, 250 × 4.6 mm i.d., 5 *µ*m	(Buffer : acetonitrile) (45 : 55) v/v	1.0 ml/min	277 nm	Good resolution 2.4	Method accepted

**Table 2:** Recovery data of the proposed HPLC method.

% spiked level	Replicate number	Peak area	% recovery	Mean %RSD
50	1	1508.4	101.9	101.5
	2	1495.5	101.0	0.43
	3	1503.5	101.6	

100	1	2950.7	99.4	99.4
	2	2950.8	99.4	0.06
	3	2953.8	99.5	

150	1	4443.5	100.2	100.1
	2	4435.4	100.0	0.13
	3	4431.9	99.9	

Mean (% of recovery)	98.0–102.0		100.318	
%RSD	Max 2.00		0.964149	

**Table 3:** System precision data from the standard solution of the proposed HPLC method.

Replicate number	RT	Peak area	Number of theoretical plates	Tailing factor
1	3.954	2952	1.32	6274
2	3.956	2951	1.36	6388
3	3.961	2951	1.35	6363
4	3.959	2960	1.33	6364
5	3.961	2953	1.36	6386
6	3.965	2946	1.36	6441
7	3.962	2949	1.38	6479
8	3.965	2950	1.35	6486
9	3.965	2954	1.35	6464
10	3.969	2958	1.33	6471

Average	3.962	2952	1.3	6412
%RSD	—	0.10	—	—

**Table 4:** Method precision data from the sample solution of the proposed HPLC method.

Alogliptin 6.25 mg tablet					
Replicate number	RT	Peak area	Tailing	Plates	% assay
1	4.025	3009	1.54	8086	99.2
2	4.024	3012	1.52	8049	99.2
3	4.027	3009	1.48	8101	99.2
4	4.027	3009	1.49	8105	98.6
5	4.028	3015	1.50	8039	99.3
6	4.027	3012	1.50	8107	99.5

Average	4.026	3011.0	1.5	8081	99.2
%RSD	—	0.1			0.31

**Table 5:** Robustness data of the proposed HPLC method.

Parameter		%RSD of standard peak area	%RSD of assay
Column temperature	25°C	0.07	0.15
	30°C (normal)	0.03	0.19
	35°C	0.04	0.2

Wavelength	274 nm	0.06	0.07
	277 nm (normal)	0.03	0.19
	280 nm	0.06	0.17

Mobile phase composition	−5% acetonitrile	0.05	0.20
	Normal	0.03	0.19
	+5% acetonitrile	0.02	0.14

Flow rate	0.8 ml/min	0.04	0.11
	1 ml/min (normal)	0.03	0.19
	1.2 ml/min	0.08	0.23

**Table 6:** Solutions stability data of the proposed HPLC method.

Parameter		RT	Avg. peak area	RSD peak area (%)	Tailing factor	Recovered (%)	Number of theoretical plates
Standard solution	0 h	4.034	3022.7	0.07	1.5	—	8058
	After 24 h at 30°C	4.035	3021.7	0.2	1.6	100.0	8143
	After 24 h at refrigerator	4.049	2983.7	0.08	1.5	98.7	8137

Sample solution	0 h	4.034	2995.7	0.07	1.5	—	8142
	After 24 h at 30°C	4.035	3001.3	0.3	1.5	100.2	8179
	After 24 h at refrigerator	4.036	3000.0	0.2	1.6	100.1	8188

## Data Availability

The data used to support the findings of this study are available from Dr. Hani shtaya (hshtaya@birzeit.edu) upon request.
